# Identifying Facilitators and Barriers to Neonatal Intensive Care Unit Visitation in Mothers of Low Socioeconomic Status: A Qualitative Investigation

**DOI:** 10.3390/children11111390

**Published:** 2024-11-16

**Authors:** Dana B. McCarty, Renée M. Ferrari, Shelley Golden, Bharathi J. Zvara, Wylin D. Wilson, Meghan E. Shanahan

**Affiliations:** 1 Division of Physical Therapy, Department of Health Sciences, School of Medicine, University of North Carolina at Chapel Hill, Chapel Hill, NC 27599, USA; 2 Department of Maternal and Child Health, Gillings School of Global Public Health, University of North Carolina at Chapel Hill, Chapel Hill, NC 27599, USA; 3 Department of Health Behavior, Gillings School of Global Public Health, University of North Carolina at Chapel Hill, Chapel Hill, NC 27599, USA; 4 Theological Studies Division, Duke Divinity School, Duke University, Durham, NC 27708, USA

**Keywords:** neonatal intensive care unit, mothers, visitation, socioeconomic status, preterm infants

## Abstract

Background/Objectives: The experience of parenting in a highly medicalized, unnatural environment can result in impaired mother–infant bonding, but increased maternal presence at the infant’s bedside has been associated with improved infant and maternal outcomes. The primary objective of this study was to explore barriers and facilitators during the NICU Experience in regard to maternal presence in an NICU. Methods: We interviewed 12 mothers (7 Black, 5 white) of low socioeconomic status (SES) whose preterm infants (average birth gestational age of 27 weeks) were currently hospitalized in an NICU. We engaged the NICU Family Advisory Board in all steps of the research process. Results: Barriers and facilitators to maternal presence spanned all levels of the Socioecological Model; however, barriers were mostly at the societal, community, and institutional levels, while facilitators varied based on interpersonal and individual-level factors. Assets that mothers accessed to facilitate visits, such as free housing and shuttle services, were not available to all mothers based on individual circumstances (e.g., caregiving responsibilities). While a few mothers identified negative interactions with health care practitioners, these encounters were not attributed to racism or described as barriers to visitation. Conclusions: Hospitals can support families with infants in an NICU by providing free or inexpensive short-term sibling support, alleviating the burden of parking costs, and communicating early and frequently about available institutional resources during the hospital stay.

## 1. Introduction

One in 10 infants are born prematurely (<37 weeks gestation) in the United States annually [1]. Infants born very preterm, defined as <32 weeks birth gestational age (BGA), are at the highest risk of death and developmental concerns, requiring long-term care in a Neonatal Intensive Care Unit (NICU) [2,3]. The mother’s frequent presence in the NICU can provide unique infant benefits, including improved infant physiologic stability and reduced infant stress [4], enhanced quality of infant–parent attachment [5], and improved physical, cognitive, and psychosocial development [6,7]. Higher rates of parent presence have also been associated with significantly better infant neurobehavior at term age [8]. High levels of parent engagement, defined as a parent’s self-motivation to set goals and to utilize informational resources about the unique care necessary for their child [9], along with support from the nursing and medical staff, are associated with improvements in both maternal and infant outcomes.

However, while “self-motivation”, along with support for the mother, may be a strong motivator to be present and engaged [10], mothers of infants in the NICU have described navigating numerous practical and logistical challenges to be with their infant [10,11]. Previously reported challenges to parent visitation have been linked to maternal sociodemographic factors such as transportation limitations [11,12], lack of childcare [12], hospital parking costs [10,11], lack of employment leave [12], number of children residing in the mother’s home [11,12,13,14,15], having public insurance [15], single marital status [11,15], and being of a younger age [8,11].

Previous studies have found an association between maternal race and NICU visitation rates. Greene et al. [11] and Pineda et al. [8] found that Black mothers visited their preterm infants less frequently than mothers from other racial backgrounds. Greene et al. [11] inferred, that because the majority of Black mothers in their study had public insurance (81%), SES might have also influenced maternal visitation rates; however, beyond social and economic challenges, Black individuals have also reported a lack of trust in large health care systems due to individual and collective experiences of interpersonal, institutional, and structural racism [16,17]. In NICUs specifically, Witt et al. and colleagues found that mothers of Black infants felt that racism led to lower-quality care as compared to other infants and described racist rule enforcement and resource allocation practices by the NICU staff [18,19]. These collective experiences of injustices may impact Black mothers’ presence, comfort, and trust levels in NICUs [16].

While living distance from the hospital has been observed to have varying degrees of impact on rates of parent presence [8,11,20,21,22,23], living in a rural area is associated with lower access to high-quality health care services [24]. The state of North Carolina has the second highest rural population in the nation, with about 30% of the population living in areas with less than 5000 residents or less than 2000 housing units [25,26]. Ninety percent of Level IV NICUs, which provide the highest levels of care, are located in urban areas of the US [27], yet, the majority of US families, especially those that live in mostly rural states, live more than 50 miles from these regional care facilities [28]. Care at Level IV NICUs is associated with lower mortality rates in very preterm infants [27], but distance between Level IV NICUs and maternal home address may impose a barrier for many mothers who desire to visit their infant frequently.

Evidence suggests that individuals who inhabit multiple forms of minoritized identities at the intersections of race and class experience the greatest barriers to accessing health care services while also experiencing disempowerment in the health care system [29,30,31,32]. The theory of intersectionality considers the multiplicative effects of multiple categories of social group membership [33] and that marginalized groups may face the greatest barriers in regard to being by their infant’s bedside [31].

While previous research provides evidence to suggest that race and SES influence maternal presence in NICUs, a better understanding of why these challenges exist is a crucial step towards developing effective interventions to improve outcomes among NICU families [34]. Research also is needed to identify the facilitators or assets that families use to improve both health care access and health outcomes so that these resources may be leveraged, developed, and expanded [35]. Analysis of sociodemographic factors alone does not fully account for observed differences in parent NICU visitation rates; therefore, a qualitative examination of mothers’ unique lived experiences is needed to provide insight into the associations between sociodemographic predictors (race and SES) and parent presence.

Our analyses are unique because of the application of intersectionality theory in the interpretation of the data. Based on the theory of intersectionality, we anticipated that Black mothers of low SES would report greater challenges regarding NICU visitation than white mothers of SES and that distance lived from the hospital would also increase reported challenges to visitation. Our approach to this project is also unique because we collaborated with community partners throughout the study with the goal of generating meaningful recommendations that, if applied, would benefit mothers who wish to be with their infants in the NICU. By engaging theoretical application and novel approaches with intent to center mothers who are often marginalized in the NICU setting, we have worked to reveal important considerations for facilitating maternal presence in the NICU so that infant and maternal health can be optimized.

The primary objective of this research was to explore individual, interpersonal, institutional, community, and society-level barriers and facilitators to maternal presence in the NICU through semi-structured interviews with mothers of low socioeconomic status (SES), representing both white and Black races, to better understand diverse challenges and assets that prevent or support NICU presence. The secondary objective of the project was to collaborate with community partners to interpret findings and establish recommendations to benefit the communities being examined.

## 2. Methods

### 2.1. Participants and Setting

The study was conducted in the Newborn Critical Care Center at the University of North Carolina Children’s Hospital, a Level IV intensive care nursery that treats over 800 infants annually, with approximately 150 of those infants being born very preterm or <32 weeks gestational age. The study was approved by the University of North Carolina at the Chapel Hill Institutional Review Board in April of 2023.

All infants were admitted into a 60-bed NICU with “pods” or rooms, accommodating up to 6–10 infant beds. Each bedspace could be separated by curtains for privacy and could accommodate up to 2 chairs. Parents could visit at all hours of the day and night but were not permitted to stay overnight. When the infant medical status improved enough to begin working on feeding by mouth, infants were transferred to a single room in the NICU step-down unit.

Mothers were eligible to be interviewed if their infants were currently hospitalized, received Medicaid (proxy for low SES), were of Black or white race, were born at ≤32 weeks gestational age, and were approaching term-equivalent age. This age requirement was selected to ensure that mothers had experienced several weeks of infant hospitalization by the infant’s term-equivalent age. The maternal inclusion criteria were that the mother was the infant’s biological parent, that they were able to speak and understand the English language, and that must be at least 18 years of age. Mothers were excluded if their infant currently required sedation and/or mechanical ventilation, if their infant did not have plans for imminent hospital discharge, if their infant died, or if they did not have custody of the infant. These exclusions were made to ensure similar lengths of stay and infant medical trajectories at the time of interview. Standard Reporting for Qualitative Research [36] guidelines were used throughout the research process.

### 2.2. Procedures

The Primary Investigator (PI; DM) screened for eligible infants biweekly to identify eligible parents. The PI called the mother to discuss interest in the study and then obtained consent over the phone or at a scheduled in-person visit. Once consent was obtained, the PI scheduled an interview with the mother either in-person or over the phone, per the mother’s preference. All mothers who completed interviews were provided with a USD 50 gift card upon interview completion (provided in-person or mailed to home address).

In-person interviews were conducted by the PI at the hospital in a private room or at the infant’s bedside with the curtains pulled to maintain privacy. All demographic data (race, infant Medicaid status, maternal age, and home address) were collected from the infant’s medical chart. Semi-structured interview questions were developed based on current evidence for facilitators and barriers to NICU presence [8,11,12]. The questions addressed the mother’s experiences in the NICU, interpersonal interactions with the NICU staff, and the logistical steps and challenges navigated when planning NICU visitation (including distance traveled and resources necessary). Mothers were asked about inclusion in medical decision-making and about the qualities of trustworthy health care practitioners (HCPs), as these two concepts have been linked to structural and institutional racism [37,38]. Trustworthiness, in the context of health care, is a subjective and interpersonal judgment made by the patient that may be enhanced by a HCP’s ability to communicate competence and care towards them and that may be complicated by a power differential or lack of racial concordance between a HCP and patient [39]. Finally, all mothers were asked if they experienced bias or felt “judged” during interactions with the HCPs they encountered. Interviews lasted an average of 31 min (range: 12–57 min) and were audio-recorded and transcribed for analysis. See Appendix A for the semi-structured interview questions.

### 2.3. Community Engagement

Study procedures and the semi-structured interview guide (Appendix A) were presented to the UNC NICU’s Family Advisory Board (FAB), made up of parents with infants who were previously in a NICU for input prior to study initiation. Additionally, the semi-structured interview guide was pilot tested with one Black member of the FAB who provided in-depth feedback about the wording of questions, approaching mothers, and cultural considerations for conducting the interviews.

Following the study period, codes and subcodes from the maternal interviews were presented and solidified in consultation with the FAB. The PI engaged the FAB in a focus group discussion to develop recommendations to improve maternal presence in the NICU through incorporating their own personal experiences, their knowledge of available hospital resources, and interview results.

### 2.4. Data Analysis

A combination of directed content analysis and thematic analysis was used to analyze the qualitative data [40]. In the context of this study, evidence from previous work examining maternal presence in the NICU contributed to the development of the semi-structured interview questions and subsequent provisional, or researcher-generated, codes. After the initial coding analysis, the primary coder (DM) developed subcodes to provide additional details about categories and patterns observed [41] with representative in vivo and direct quotes. A secondary coder (KBA) reviewed each code and subcode and identified additional quotes from participant transcripts for additional insight and quality.

Following initial coding, the primary coder performed a secondary review of the dataset using a thematic analysis to describe broad categorical and phenomenological themes across established codes [41]. Co-authors (SG, RF, BG, WW, and MS) reviewed a selection of codes, subcodes, and quotes to verify broad themes established by the primary coder. NVivo version 14 was used to manage the data [42].

## 3. Results

### 3.1. Sample

Of 23 eligible mothers, 3 declined participation, 6 did not respond to phone calls or messages, and 2 infants were too sick at the age of eligibility. We enrolled 12 mothers, 5 white and 7 Black, whose average age was 29.7 years (range 20–39), who had infants with an average BGA of 27 weeks (range, 23–32), and who, at the time of the interview, had infants with an average length of hospitalization of 9 weeks (range, 3–13 weeks). The mothers’ home addresses were an average of 57 miles (range, 33–90) from the hospital. Six of 12 mothers were staying locally at the Ronald McDonald House (RMH), a local charity that provides free lodging for families of children hospitalized at the UNC Medical Center [43]. See Table 1 for full reporting of maternal characteristics.

### 3.2. Analysis

A total of 8 codes, 7 a priori and 1 emergent, and 30 subsequent subcodes were developed for the dataset (Table 2). The a priori codes included barriers to maternal presence, facilitators to maternal presence, NICU experience, interpersonal interactions, trustworthiness, shared medical decision-making, and bias. The additional code of Emotions was added, as the primary coder noted patterns of positive and negative perceived emotions through in vivo quotes.

**Figure 1:**
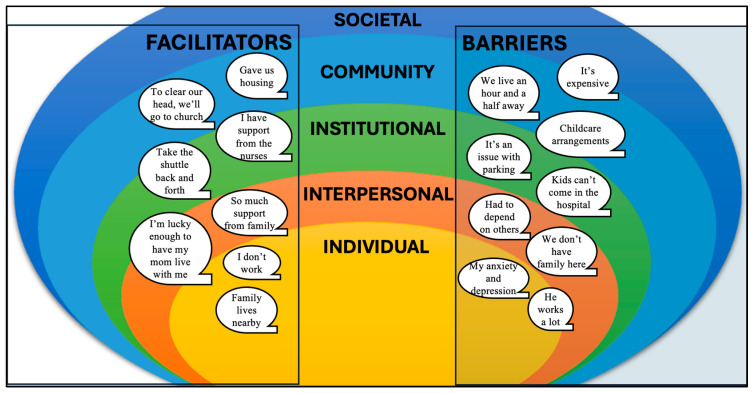
Theme 1—Facilitators and Barriers spanning the Socioecological Model.

**Figure 2:**
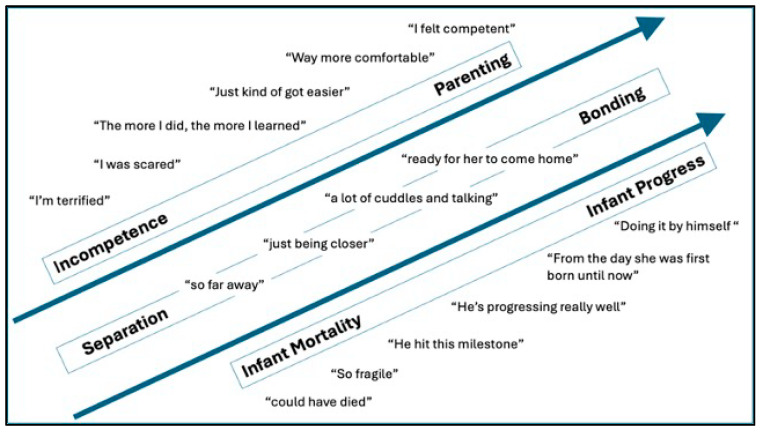
Theme 2—positive trajectories across the NICU experience.

Two broad themes emerged from secondary analysis of the dataset. A secondary analysis of Barriers and Facilitators revealed that both descriptive and in vivo quotes spanned from individual-level differences to systemic or societal levels of influence. In other words, Barriers and Facilitators spanned all levels of the Socioecological Model (SEM) [44] (societal, community, institutional, interpersonal, and individual (Figure 1)) and was identified as the first theme. Next, mothers often described their experience in terms of how it related to the timeline of events in the NICU. Six subcodes falling under the code “NICU Experience” demonstrated a positive trajectory over time, such that mothers expressed more negative and less-developed perceptions of themselves and their infants immediately after birth, but perceptions became more positive and developed towards the present day (Figure 2) [41]. As a result, the second theme was identified as “Positive Trajectories Across the NICU Experience”.

#### 3.2.1. Barriers to Maternal Presence

Mothers described the resource, time, and logistical constraints that prevented daily hospital visitations. Subcodes identified under this theme included distance of commute, resource strains, institutional barriers, responsibilities outside of infant care, and mother’s health and recovery. Barriers spanned all levels of the SEM, with a greater concentration being found in societal, community, and institutional levels. Societal barriers included resource strain and distance of commute. Most mothers lived at a considerable distance from the hospital in rural communities with generally more affordable housing (societal-level barrier) [45] but without Level IV neonatal intensive care (community-level barrier) [27]. Institutional barriers included parking logistics, parking costs, and hospital visitation policies that increased time and effort in terms of visitation and placed limitations on the presence of family members. The inability to stay overnight while the infant was in the NICU denied parents both accommodation and the ability to learn to care for their infant around the clock. Mothers reported that these logistics were even more difficult to navigate in the early stages of birth recovery, when dealing with mental health challenges, when caring for other children at home, or when returning to work outside of the home. Seven of the 12 mothers had children at home and had to plan NICU visits when they could arrange childcare or during school hours.

*“I know they mentioned to me there was a [parking] pass or something I could get…And then once I was able to bring myself, I was able to come as I could in between the other children being in school and have to be picked up and doctor’s appointments and all that good stuff. So, I have to come in between all of that going on?”*

*(Mom 5)*

#### 3.2.2. Facilitators to Maternal Presence

Mothers discussed various supports and assets that made their presence in the NICU possible. While facilitators spanned all levels of the SEM, most facilitators were interpersonal or individual-level factors like social supports, job flexibility, resource provision, and housing/accommodations near the hospital. Several mothers talked about their partners or parents who were involved in the infant’s care and shared some responsibilities for visiting the infant. Others had flexibility in visitation because their spouse or parents were employed and able to support them financially. Free accommodations at the Ronald McDonald House (RMH) or staying locally with family members made daily hospital visits attainable for mothers who lived further away; however, the RMH was not an option for mothers who were working at the time or who had other children. Some mothers expressed gratitude once their infant had been transitioned from pod-based care to a single room, which allowed the mother to stay overnight with the infant.

*“And I do realize that I’m in a unique circumstance where I am a stay-at-home parent and my mother, she doesn’t have a formal job…So, I have family to watch my kids and I’m flexible with time. That is not the case for the majority of parents I would say.”*

*(Mom 1)*

*“I am driving, but just to save with money and parking in the parking garage, I do take the shuttle back and forth. That’s one of the best things honestly.”*

*(Mom 11)*

Mothers accessed resources that facilitated their NICU visitation differently based on different factors at interpersonal and individual levels of the SEM. While important institutional and community-level facilitators like RMH and staying overnight in the infant’s room were identified, some mothers could not access these resources based on individual- and interpersonal-level factors (e.g., other children at home, limited family support, work responsibilities).

#### 3.2.3. NICU Experience

Individual factors contributed to a mother’s desire and ability to be at the infant’s bedside as well. Mothers described both negative and positive perceptions of the NICU stay, with many negative experiences occurring early after birth and more positive experiences occurring over time. Negative subcodes included mortality, infant size, separation, and incompetence (see Table 2 for definitions of subcodes). Even for individuals with parenting experience, mothers acknowledged that the risk of complications and the variable infant progress day to day led to uncertainty about how to interact with their infant, especially in the early days of hospitalization. Mothers also lamented being separated from their infant and missing important developmental milestones.

*“You sit there and wonder when he’s coming home, when he’s coming home. And for us, there’s been moments where we think we can start to see the finish line, and inevitably something happens that prolongs his stay even more.”*

*(Mom 12)*

*“…you miss a lot of firsts. You miss the first holding him. You miss the first diaper change. You miss his first outfit. You miss a lot of stuff.”*

*(Mom 1)*

Positive subcodes included bonding, parenting, and infant progress (Table 2). Mothers described their individual perceptions along a timeline, which often evolved throughout the NICU stay and moved along a trajectory from negative to positive (Figure 2).

*“So, going from one pound to five pounds is just amazing. I’m watching her grow outside my womb. It’s just amazing. It’s amazing. It really is.”*

*(Mom 10)*

*“Well, at first, really, all I could really do was hold her because she was just so small. She couldn’t do anything. But, now, I’m able to feed her, hold her, dress her, change her…So, now, it’s a little bit better now.”*

*(Mom 9)*

#### 3.2.4. Interpersonal Interactions

Mothers expressed a range of positive and negative interactions with health care providers that influenced their presence in the NICU. Positive interpersonal interactions were subcoded as follows: communication, encouraged infant interactions, and reassurance (see Table 2 for definitions). Mothers appreciated open, honest, and frequent communication about their infant’s progress delivered in parent-friendly language; however, true comfort at the infant’s bedside seemed to develop with encouragement to be involved in infant interactions and reassurances from HCPs. Mothers also appreciated being encouraged by HCPs to be involved and perform care activities with their infant, and they valued reassurances that they were engaging with their infant appropriately.

*So, that’s why I was saying those nurses were giving me confidence. They were just like, “Come on, we’re here. We’re not going to let nothing happen. We’ve got monitors. We can stay in here and watch you. Whatever is comfortable for you.”*

*(Mom 10)*

*…the nurses are there in person telling me, “Listen, this is normal. It happens. We’re here to help.” And they made me feel so much better.*

*(Mom 11)*

Negative interpersonal interactions were not described as often as positive interactions. Negative subcodes were characterized by disagreements about care, poor communication, discouraged infant interactions, and feelings of guilt or being a burden. Mothers expressed frustration when HCPs did not respond to their concerns about how their infant should be cared for or when communication was inconsistent. At times, mothers reported feeling like they did not want to trouble the nurses with requests to hold or interact with their infant and felt guilty or like a burden if they had to ask the nurses for help.

*They will not fully change his dressing. All they do is wipe off the little plastic piece that they put over it….That can cause him to get an infection. That’s what worries me.*

*(Mom 7)*

*“Why did no one call me when my child moved? I haven’t had an update in four days. What’s happening?”*

*(Mom 1)*

Mothers also felt that HCPs would take over certain care activities if they felt the mother was performing something incorrectly. Mothers felt guilty for asking to hold or interact with their infant if they could not visit for long periods of time.

*Well, it kind of depended on the nurse because some are like because he was connected to so much, you know, they prefer that you’re able to stay, you know, an hour or more. I mean, sometimes let’s face it, I can’t stay as long*

*—(Mom 5)*

Finally, mothers reported that poor communication caused them to miss out on taking advantage of certain resources that would have increased their capacity to visit. Mothers expressed confusion about how to obtain a medical referral for free housing at the RMH or how to obtain a weekly reduced rate parking pass, and they also reported that their infant had been hospitalized for several weeks before they learned of such resources.

*I asked them about [free housing] a couple of months ago right after I had him. I went home because they didn’t let me know anything about this at the hospital.*

*(Mom 7)*

#### 3.2.5. Trustworthiness

We also sought to explore mother’s perceptions of trustworthiness in HCPs. Mothers were asked the following question: “When you think of the health care practitioners that you’ve engaged with, who comes to mind as trustworthy? What are the qualities that make them trustworthy?” Mothers described trustworthy HCPs as ones who demonstrated care for the infant, established a connection with the parent, and created a sense of mutual trust. Trustworthy HCPs were often described as individuals who cared for patients and families beyond necessary job requirements and who sought to establish rapport with parents.

*“While he’s talking to me, he’s rubbing [the infant] on the head, just being very personable like he cares for this little baby. That eases me so much.”*

*(Mom 3)*

*“It’s the way they talk and they actually let me hands-on and help.”*

*(Mom 7)*

*“There are people who are on top of it, and there are people who are on top of cares and on top of feeding where they don’t make it feel like a chore.”*

*(Mom 4)*

HCPs were considered untrustworthy for a variety of reasons that were often linked to the parent’s experience of HCPs acting unprofessionally. These experiences including parents witnessing HCPs speaking negatively about another parent and the HCP not responding promptly to their concerns. HCPs were even considered untrustworthy if they showed little interest in communicating updates to parents.

*“So, that can kind of make you lose trust and confidence in somebody if you hear them complaining about a parent when they’re not there.”*

*(Mom 2)*

#### 3.2.6. Emotions

The “emotions” theme emerged as mothers described a range of feelings towards their infant, HCPs, and the NICU setting. Many named emotions could be mapped along the trajectories moving from negative to positive (Figure 2). Mothers sometimes described being caught off guard by their emotions and recognized how emotions played a role in their interactions with the infant and HCPs.

*“Yeah. You want to comfort, you want to soothe, but you can’t. You can’t do anything. All you can do is just watch her through a glass and she has to fight for herself.”*

*(Mom 2)*

*“I think the first time I held her, she was like maybe two weeks old, because I was scared to hold her. There was no way you could hold her. I was like, “I am scared.’”*

*(Mom 10)*

#### 3.2.7. Shared Medical Decision-Making

Most mothers felt that they were included and welcomed into the infant’s medical care. The degree to which each mother described her inclusion was often related to how much the mother initiated engagement with the care team. Many mothers reported asking follow-up questions, taking notes, and being very interested in details of their infant’s care, while, in turn, HCPs acknowledged the mother’s role and input about the infant’s care. Other mothers reported that they felt comfortable with the amount of information and explanations the medical team shared with them.

*The way she talked, wasn’t like, ‘I’m an expert and I know better’….It was more like, ‘Okay…Tell me what you think and I’ll tell you my professional opinion.’ They were like, ‘We’ll give you guys time. We’ll give her time, and once time runs out, then we’ll start discussing it.’”*

*(Mom 2)*

*“But as far as I’m not comfortable with this or that, I don’t think they really care. They’re like, ‘Well, we’re going to do it anyway because it’s what he needs. But we want you to know why.’”*

*(Mom 3)*

#### 3.2.8. Bias

Overall, mothers reported positive, supportive interactions with HCPs during their NICU stays. All mothers were asked the following question: “Did you ever feel your judged during these interactions (with your baby’s healthcare provider)?” Black mothers were also asked the following: “Do you feel your race played a role/had something to do with how these interactions went?” Of the seven Black mothers interviewed, none reported perceptions of racially biased interactions.

*“I’m trying to think. Pretty much everybody on her care team is the opposite color….Definitely not. I’ve never felt that way.”*

*(Mom 2)*

*“No, I don’t think I’ve ever experienced anything negative. I was told from one nurse…that this is a hospital [that] cares so much about mothers, especially Black mothers.”*

*(Mom 11)*

A few mothers felt judged based on perceived incompetence, age, or appearance. All mothers agreed that biased interactions were far less frequent than positive interactions, but these few interactions had a lasting impact on their comfort level in the NICU.

*“…there was one nurse in particular that kind of made me* *—kind of belittled me, I felt like, as a mom.”*

*(Mom 12)*

*“…felt like some of the older staff, like more towards grandparents age, they seem to talk down on the younger parents. I’ve had quite a few dirty looks when I take down my mask because I have a good amount of piercings.”*

*(Mom 4)*

### 3.3. Community Engagement: Family Advisory Board Collaborative Recommendations

In response to preliminary results from the maternal interviews and their own experiences, the FAB considered ways to (1) develop partnerships between existing resources, (2) raise awareness about existing resources available to NICU families, and (3) identify resources that need further development in order to be readily accessed and supportive. In consultation with the FAB, the following three institutional recommendations to improve maternal access to their infants in neonatal intensive care were created.

Recommendation 1: Hospitals should provide free or inexpensive short-term sibling support. The financial cost of childcare and the burden of making childcare arrangements was problematic for the mothers in our study. The FAB discussed that volunteers under the direction of the Child Life Specialist program at the hospital used to provide volunteer-staffed, free childcare on-site for siblings while mothers visited their infant in the NICU; however, this program ceased operations during the COVID-19 pandemic and has not been started again. They recommended that future efforts to provide sibling support should be financially supported by the hospital with reliable, consistent coverage.

Recommendation 2: Hospitals should alleviate parking burdens for families whose infants require long-term hospitalization. Both mothers in the study and FAB members expressed frustration with parking costs and inadequate resources by the hospital to alleviate these burdens. For example, a FAB member reported that hardship forms are available for parents who qualify for free parking based on low income but that parents must go to the parking attendant or parking offices to request the form in-person. Parents are also offered less expensive weekly parking passes for remote lots; however, parking at a distance adds time to their commute and creates logistical challenges when attempting to schedule arrivals and departures based on shuttle schedules.

Recommendation 3: Parents would benefit from early, frequent, and consistent communication from staff members about institutional resources during NICU stays. Mothers and FAB members commonly reported learning about resources available to them several weeks into their infant’s hospitalization and often reported learning about resources “by chance” instead of through modes of direct inquiry. FAB members reported that many instructions for accessing resources are posted throughout the NICU but that mothers do not see them or do not know that they are eligible to use them. FAB members also acknowledged amidst the stress of the NICU environment that they often did not fully absorb all the information provided to them and needed considerable repetition to remember or access that information. Finally, some resources, like weekly parking passes, are only options for families whose infant is hospitalized for longer than 2 weeks, and parents are likely forget about these options; therefore, the FAB members suggested that families should be reminded when they have reached this point of eligibility and be provided with the necessary information, again, if necessary, to access the resource.

## 4. Discussion

Mothers reported challenges to their presence at their infant’s bedside as well as the strategies they used to overcome them. Barriers and facilitators to maternal presence spanned all levels of the SEM. Barriers were mostly at societal, community, and institutional levels. Facilitators varied based on interpersonal and individual-level factors; assets that mothers accessed to facilitate visits, such as free housing and shuttle services, were not available to all mothers based on individual circumstances (e.g., caregiving responsibilities). While a few mothers identified negative interactions with HCPs, these encounters were not attributed to racism or described as barriers to visitation.

Common barriers discussed were in alignment with previous study findings. Parking was an institutional barrier that was frequently mentioned because it caused considerable planning and financial strain. Lower cost parking options were available for a remote lot, but some parents reported that they found it difficult to purchase these passes or had trouble understanding the shuttle pick-up schedule from the remote lot. Studies documenting parking as a barrier to general health care access are limited. However, this unmet need for affordable parking has been documented for NICU families [10,11] and the families of oncology patients [46,47], highlighting the high frequency of visits and caregiving roles commonly seen in these populations.

While having access to free parking during the NICU stay has been identified as an important need for NICU families [10,11,48], free parking interventions may not be enough to address the multiple downstream effects of low socioeconomic status. For example, Northrup et al. found that a free parking trial did not improve parent visitation rates in the NICU as compared to controls. Instead, they found that higher parent income, access to a car, and fewer children were all positively associated with parent visit rates [49].

Andrews et al. evaluated the effect of a non-conditional financial transfer of USD 200/week to mothers of infants in the NICU living >30 miles from the hospital in terms of maternal presence and engagement [50] and found that mothers who received financial transfers had higher rates of skin-to-skin holding, visitation, and breastmilk provision than controls. The authors posited that, unlike free parking, a cash transfer may have been used in various ways to support mothers’ ability to be present in the NICU, allowing families facing a multiple resource-related challenges to have flexibility in addressing their needs [50].

Distance traveled was a barrier that many mothers had to overcome, but this challenge was mitigated for half of our study sample by staying locally at the Ronald McDonald House (RMH). Families qualify to stay at the RMH if their permanent residence is >35 miles away from the hospital, the families are directly involved in the child’s care, and a medical referral is received [43]. Siblings are allowed to stay at the RMH, but they are not allowed to visit the hospital; therefore, one caregiver must be responsible for the sibling at all times, as childcare is not provided [43]. As a result, mothers with children in school or without family to care for the sibling during hospital visits cannot take advantage of RMH accommodations. While families in our study were grateful for the parking and housing resources that they learned about from the hospital, many families expressed frustration about poor communication delaying their access to free accommodations.

Thiele and colleagues identified a 12% increase in daily NICU visitation for families who stayed at a local RMH as compared to controls [51]. Because it removes distance and parking barriers, use of an RMH may result in higher visitation frequencies for families living further distances from the hospital than free parking alone. The requirement for daily visitation while staying at the RMH likely also contributes to high visitation rates. While no parents in our study expressed feeling overwhelmed or burdened by daily visitation requirements during the RMH stay, this has been expressed by RMH guests in other studies [10]. Mothers also described increased access to their infant once the infant had transferred out of the pod-based intensive care unit and into a single room on a step-down unit. In contrast to the pod-based NICU, parents could stay overnight in single family rooms, which include pull-out beds, showers, and free meals for breastfeeding mothers.

No Black mothers (n = 7) in our study reported feeling judged based on race during interactions with HCPs. This finding could be attributed to multiple factors, including a lack of racial concordance with the interviewer (white female) [52], which may have limited Black mothers’ honest portrayals of microaggressions and stereotyping. A recent report that was conducted over 100 interviews and 18 focus groups with Black Californians revealed that two-thirds adjust speech and/or behavior when speaking with a HCP to make the HCP more comfortable [53]. Other qualitative studies have documented that Black mothers in the NICU were discouraged from performing skin-to-skin care [54], and in a large-scale qualitative study that interviewed parents representing 32 east-coast NICUs, Black mothers had lower rates of parent satisfaction as compared to white mothers [55]. Three mothers in our study, however, did report feeling judged based on their young age, their appearance (e.g., tattoos, piercings), and their competency in caring for their infant, which is consistent with other qualitative reports of maternal experiences in the NICU [56].

It is possible that we did not identify challenges unique to the intersection of race and SES for a variety of reasons. First, it is important to distinguish the difference between social class and socioeconomic status, as the two are often used interchangeably despite their meanings being conceptually different. Socioeconomic status is based on objective measures such as income and occupation [57]. Social class comprises SES and subjective social status, or an individual’s perception of their social class in relation to others [58]. In contrast to the theory of intersectionality, Macrae describes an alternate approach to how humans deal with social complexity called the “category dominance model.” [59]. In this model, “when facing multiply categorizable targets, this view holds, people will often rely on a single social category to guide social perception.” [60,61]. Because infants in the NICU require a certain level of competence to be safely handled and cared for [62,63], it is possible that HCPs in our study judged mothers primarily based on their competence in infant care. Because perceived competence is associated with higher social class [59], when operating using the category dominance model, HCPs may have defaulted primarily to class-based judgments. Results from a study by Connor et al. demonstrated a well-described phenomenon—across five separate studies examining interpersonal interactions, “pro-upper-class/anti-lower-class biases” were observed consistently, while race-related biases were less consistently appreciated [60,64,65].

First impressions of social class are made quickly, often through a stereotyped assessment of nonverbal cues, including facial expressions and attractiveness [66]. These rapid judgments have real consequences, as perceptions of higher social class are associated with better employment opportunities [66], trustworthiness [67], and health outcomes [68,69]. Therefore, while both physicians and nurses in our study have participated in required annual diversity, equity, and inclusion training programs, these trainings may focus more on objective factors like race [70,71] and SES [72], and focus less on topics that are difficult to assess and describe, like social class [68]. It is possible that the intersection between perceived social class and other social group memberships (e.g., race, gender) may have a greater influence on a mother’s presence and comfort in the NICU than proxies for SES like insurance status, which is used in our study [66].

The concepts of trustworthiness and shared medical decision-making were specifically explored to better understand the potential impact of structural and interpersonal racism as well as class bias in regard to maternal presences in the NICU. The development of trustworthiness in HCPs takes place at the interpersonal level, but repercussions from a lack of trustworthiness may be observed at the population level. Evidence suggests that Black adults experience delayed diagnoses [73], limited access to medical services, and low engagement with recommended therapies [74] as compared to other racial groups; it is also found that Black women express a lack of shared decision-making, stereotyping, invalidation, and dismissal by their reproductive HCPs [17]. Authors suggest that these experiences may lead to future self-protective actions in health care encounters, such as avoidance of the health care system or a sense of “being on edge” that perpetuates a sense of the lack of trustworthiness of HCPs [17].

Mothers in our study did not associate HCP trustworthiness or shared medical decision-making with the characterizations of racially biased interactions. This finding was in opposition to previous studies [19,75] that documented Black maternal experiences of health care-related racism at various levels of the SEM, from structural (i.e., the untrustworthiness of the health care system), to institutional (i.e., differences in resource allocation and rule enforcement) and interpersonal (i.e., neglectful and judgmental treatment, hierarchical flow of information, and power imbalances) [18,19] levels. Mothers in our study described trustworthy providers as ones who fostered a “connection” with them, displayed “compassion” towards them and their infant, and called them by name (i.e., did not use “mom”). These characteristics, which have previously been linked to parent satisfaction with pediatricians [76], may reveal that parents are fundamentally seeking comfortable interactions in an otherwise uncomfortable setting.

The theme of positive trajectories applied to many subcodes within the larger “NICU Experience” code. Mothers acknowledged positive trajectories in their infant’s growth as well as in their own growth as parents. Additionally, mothers’ emotional descriptions of interacting with their infants immediately after birth were characterized by fear and uncertainty, which was in stark contrast to their joyful emotions about interacting with their infants at their current age. These trajectories, which shifted from negative to positive perspectives, may have influenced the mothers’ capacity to be present and engaged at the bedside. Conflicting results have been noted in the literature. Greene et al. [11] found an association between higher maternal visitation rates and higher maternal anxiety, while Gonya et al. [20] found that higher levels of stress were associated with lower parent visitation rates. These opposing findings might be explained by the timing of data collection (e.g., beginning vs. end of NICU stay) regarding the trajectory of emotions and growth that parents experience during the NICU stay and alternate coping mechanisms (e.g., avoidance vs. presence) experienced at opposite ends of these trajectories.

## 5. Limitations

The sample size for this study was small; however, because inclusion criteria were created to increase the likelihood of shared experiences (e.g., infant gestational age, Medicaid status), we believe that the small sample is representative of the NICU where the study was conducted. We recognize that the limited sample reduces generalizability to other NICUs.

Because we sought to understand the influence of race but not ethnicity, we did not include Hispanic mothers in our sample; therefore, this study does not account for unique cultural or language-related challenges facing this population. There was a lack of racial concordance between the PI and Black mothers in the study, potentially creating an environment where Black mothers did not feel they could be honest about experiences of racism in the NICU. While all mothers were assured that study participation was separate from their infant’s care, many mothers may have limited their sharing of negative experiences if they were concerned it would negatively impact their infant’s care.

Finally, despite the codes and subcodes being reviewed by a secondary coder for additional insights, all interviews and codes were conducted and developed by a single researcher, which may introduce bias to the analysis. Braun and Clarke [77] support using a single coder as “good practice” in thematic analysis and do not recommend use of multiple coders to reach a “consensus” about a code. Use of a primary interviewer and coder affords deep engagement with the data, and we have limited the potential for bias in our interpretation of results through engagement with the NICU Family Advisory Board, a secondary coder, and review of data with co-authors.

## 6. Conclusions

Our findings did not fully align with intersectionality theory, which would support the idea that Black mothers of low SES would report greater challenges regarding NICU visitation than white mothers. Instead, Black and white mothers of low socioeconomic status described similar Barriers and Facilitators to their presence in the NICU. Black mothers in our study did not describe racially biased interactions with HCPs that discouraged them from visitation. It is possible that negative interactions with HCPs were more related to subjective assessments of social class than more objective measures of social group membership like race and SES. Most mothers did not live close to the hospital and navigated many societal, community, and institutional challenges related to transportation, parking, and time. Accessing resources such as free accommodations varied based on interpersonal and individual factors such as whether mothers were the primary caregiver for other children and/or if the mother had family support to facilitate long-term visits. Overall, mothers described the NICU experience along the timeline with a positive trajectory in regard to infant growth, parent sense of competence, and closeness with their infant. Hospitals can support families with infants in the NICU by providing free or inexpensive short-term sibling support, alleviating the burden of parking costs, and communicating early and frequently about available institutional resources during the NICU stay. 

## Figures and Tables

**Table 1:** Maternal Characteristics.

	Maternal Age	Maternal Race	Infant Gestational Age	Length of Hospitalization at Time of Interview	Home Address Distance in Miles from NICU	Other Children	Working Outside of the Home at Time of Interview	Partner	Access to Free Living Accommodations
Mom 1	33	W	29 w 4 d	8 weeks	38	Yes	Yes	Yes	Yes, family
Mom 2	31	B	23 w 1 d	13 weeks, 2 days	74	No	No	Yes	Yes, RMH
Mom 3	30	W	30 w 0 d	4 weeks, 4 days	76	Yes	No	Yes	No, commuting
Mom 4	20	W	27 w 0 d	6 weeks, 4 days	33	No	No	Yes	No, commuting
Mom 5	36	B	27 w 5 d	9 weeks, 3 days	39	Yes	No	No	No, commuting
Mom 6	39	B	24 w 6 d	13 weeks, 1 days	43	Yes	No	No	No, commuting
Mom 7	34	W	32 w 1 d	8 weeks, 5 days	55	No	No	Yes	Yes, RMH
Mom 8	30	B	25 w 2 d	10 weeks	90	Yes	No	No	Yes, RMH
Mom 9	21	B	26 w 0 d	11 weeks	68	No	Yes	Yes	No, commuting
Mom 10	28	B	26 w 1 d	11 weeks, 2 days	38	Yes	No	Yes	Yes, RMH
Mom 11	26	B	31 w 0 d	3 weeks, 3 days	46	No	No	No	Yes, RMH
Mom 12	28	W	26 w 6 d	11 weeks, 4 days	84	Yes	No	Yes	Yes, RMH

Key: w = weeks; d = days; RMH = Ronald McDonald House.

**Table 2:** Codes and Definitions for Dataset.

Code	Definition	Subcode		Definition	In Vivo Phrases Examples
Barriers to Visitation	Descriptions of logistical challenges and/or resource- or time-related barriers to hospital visitation	Distance of Commute		Distance traveled; time necessary to travel from home to hospital	“going back and forth”
		Resource strains		Financial strains to support costs of gas, parking, and food while away from home	“It’s expensive” “depending on gas prices”
		Institutional Barriers		Difficulty navigating parking logistics, hospital check-in, visitation policies	“it’s an issue with parking”
		Responsibilities outside of infant care		Visitation limited by work hours (either mother or mother’s partner) and caregiving responsibilities	“I had to depend on others” “depends on childcare”
		Mother’s health/recovery		Mother’s own health care appointments and/or restrictions on driving	“I was also sick” “anxiety and depression that I’ve had”
Facilitators to Visitation	Descriptions of maternal supports that make regular visitation more feasible	Family and/or social supports		Family and/or community assistance in ways that facilitate the mother’s visits	“so much support from…family”
		Job flexibility and/or resource provision		Flexibility in mother’s or partner’s work or finances that facilitate visits	“works from home” “husband works”
		Housing		Having a place to stay locally	“gave us housing” “lives nearby”
NICU Experience	Mother’s perceptions of the experience of having a preterm infant in the NICU	Positive	Bonding	Holding, cuddling, skin-to-skin care, or other developmentally appropriate interactions with infant	“ready to love on her” “that was special” “get to hold him”
			Parenting	Activities related to caring for infant including feeding and diapering	“I feed her myself, everything” “I’m like, I’m his mom, I change his diaper”
			Infant Progress	Descriptions of how far the infant has come regarding health since birth	“Look how far she’s come” “I can’t believe this is the same baby”
		Negative	Separation	Difficulty being separated from infant and/or family members	“ready for her to come home” “I’m not whole”
			Incompetence	Not sure how to care for infant or how to feel about infant	“I don’t know” “it’s just different” “you question yourself”
			Infant mortality	Concerns about infant’s risk of death or staying/becoming sick	“laying there lifeless” “so fragile” “literally, in the palm of your hands”
Interpersonal Interactions	Mother’s descriptions of conversations and interactions with health care practitioners	Positive	Communication	Descriptions of open, honest, and regular conversations with HCPs using parent-friendly language	“be straightforward with us” “don’t hold back details” “call me daily”
			Encouraged Infant Interactions	Encouragement to hold, feed, bathe infant by HCPs	“Hey, let me show you.” “they allowed me to do that”
			Reassurance	Reassured or consoled about her experience by HCPs	“reassured me that he’s okay” “This is normal. It happens.”
		Negative	Disagreement about care	Concerns about medical or care-based decisions and actions	“one nurse that I don’t like” “can’t you see what’s going on with my child?”
			Poor communication	Lack of updates, using jargoned language	“it was confusing”
			Discouraged interactions	Limitations to mother in care activities due to actions or inactions of HCPs	“not initiated by the healthcare team” “they prefer that you’re able to stay”
			Guilt or burdensome	Feeling of being a burden to the HCP, making infant situation worse	“wrong way” “I don’t belong here”
Shared Medical Decision-Making	Whether mother felt included in decision-making related to infant’s care				“they’ll explain it” “made sure that I understood” “what I say doesn’t matter”
Trustworthiness	Descriptions of characteristics that make a HCP trustworthy or not	Positive	Care for infant	HCP going above and beyond; examples of care and compassion	“show compassion” “call them by name”
			Connection	Positive relationship between mother and HCP	“great connection” “talk as a friend”
			Mutual Trust	HCP extends trust to mother and, in turn, mother trusts HCP	“engage yourself with parents”
		Negative			“lose trust and confidence” “I don’t know” “kind of skeptical”
Bias	Perceptions of biased interactions with HCPs	Race-related		Descriptions of perceived bias related to Black race	“cares so much about mothers” “really good and safe”
		Other		Descriptions of perceived bias based on other aspects of mom’s identity	“belittled me” “dirty looks” “won’t let you help out”
Emotions	Maternal descriptions of emotions towards infant, NICU setting, or HCPs	Positive		Generally positive emotions and/or expressions of gratitude towards infant, NICU setting, or HCPs	“so happy” “really touched my heart” “it really touched me” “just being closer… kiss them” “that’s a blessing”
		Negative		Negative emotions towards infant, NICU setting, or HCPs	“it’s scary” “you can’t do anything” “really nervous” “I was sad”

## Data Availability

The data presented in this study are available on request from the corresponding author due to patient privacy and identifiable information within transcripts.

## Appendix A. Semi-Structured Interview Guide

**Semi-structured questions**
*Opening Question*
*I am going to talk with you about your overall experience visiting your baby in the NICU.*
*Your baby has been in the hospital for X months, right? What has this experience been like for you?* - *What’s been one of the hardest parts? Feel free to share a story.* - *What’s been one of the best parts? Feel free to share a story.*
*Structural and Institutional Barriers and Facilitators*
*Let us talk about your visits. First, before we even get to your time with your baby, could you describe how you get to the hospital?* - *Probe: mode of transportation, anyone give you a ride, have to take off work, etc.* - *How long did it take you to get here today?*
*How has it been for you to be separated from your baby?*
*How long is your commute to the hospital?*
*What were the things that made difficult to visit your baby on a regular basis?*
*In times that you did get to see your baby and it didn’t feel difficult, what made those visits easier?*
*Individual Perceptions*
*What are your visits like?* - *What are some of the things that make your visits difficult?* - *What are some of the things that make your visits better?*
*What kinds of things do you do when you visit your baby?* - *What are some of your favorite things you get to do with your baby?* - *Least favorite things?*
*Would you say you feel comfortable doing these activities (eg., diapering, feeding, holding)?* - *How has your comfort changed since your baby was first born?* - *When do you feel like it’s helpful for you to be here? When do you feel like it’s less helpful? Why?*
*Do you feel like a member of your baby’s medical team?* - *Do you feel like your suggestions or ideas are taken seriously?* - *Do you feel listened to?*
*Interpersonal Interactions*
*I am going to talk with you some about your interactions with different health care practitioners/clinicians that you’ve met in the hospital…*
*Since you’ve been here with your baby in the hospital, what kind of health care practitioners/clinicians have you met?* - *(prompt with nurses, physicians, therapists, social workers if necessary)*
*Of the different health care practitioners you have met, which ones have talked with you about how things are going with your baby, how to care for your baby, etc.?* - *In other words, who do you think has tried to teach you some things about your baby and care for your baby?*
*What kind of things have health care practitioners talked to you about or taught you during your baby’s hospitalization?* - *Which ones were helpful? Why?* - *Which ones were not helpful? Why?* - *Did you ever feel your judged during these interactions? If Black mother: Do you feel your race played a role/had something to do with how these interactions went?*
*Have you had an experience with a health care practitioners/clinicians where you didn’t have all of your questions answered?* - *Feel free to share the story of that experience.* - *Did you ever feel your race played a role/had something to do with how these interactions went?*
*When you think of the health care practitioners/clinicians you’ve engaged with, who comes to mind as trustworthy? Feel free to think of more than one person.* - *What are the qualities that make them trustworthy?*
*If you could tell your baby’s health care practitioners/clinicians anything you would like for them to know about how to better interact with you and/or other mothers like you, what would it be?*
